# QTL Mapping by Whole Genome Re-sequencing and Analysis of Candidate Genes for Nitrogen Use Efficiency in Rice

**DOI:** 10.3389/fpls.2017.01634

**Published:** 2017-09-21

**Authors:** Xinghai Yang, Xiuzhong Xia, Zongqiong Zhang, Baoxuan Nong, Yu Zeng, Faqian Xiong, Yanyan Wu, Ju Gao, Guofu Deng, Danting Li

**Affiliations:** ^1^Rice Research Institute, Guangxi Academy of Agricultural Sciences Nanning, China; ^2^Cash Crops Research Institute, Guangxi Academy of Agricultural Sciences Nanning, China; ^3^Biotechnology Research Institute, Guangxi Academy of Agricultural Sciences Nanning, China; ^4^Guangxi Crop Genetic Improvement and Biotechnology Laboratory, Guangxi Academy of Agricultural Sciences Nanning, China

**Keywords:** rice, nitrogen use efficiency, whole genome re-sequencing, QTL, candidate genes

## Abstract

Nitrogen is a major nutritional element in rice production. However, excessive application of nitrogen fertilizer has caused severe environmental pollution. Therefore, development of rice varieties with improved nitrogen use efficiency (NUE) is urgent for sustainable agriculture. In this study, bulked segregant analysis (BSA) combined with whole genome re-sequencing (WGS) technology was applied to finely map quantitative trait loci (QTL) for NUE. A key QTL, designated as *qNUE6* was identified on chromosome 6 and further validated by Insertion/Deletion (InDel) marker-based substitutional mapping in recombinants from F_2_ population (NIL-13B4 × GH998). Forty-four genes were identified in this 266.5-kb region. According to detection and annotation analysis of variation sites, 39 genes with large-effect single-nucleotide polymorphisms (SNPs) and large-effect InDels were selected as candidates and their expression levels were analyzed by qRT-PCR. Significant differences in the expression levels of *LOC_Os06g15370* (peptide transporter PTR2) and *LOC_Os06g15420* (asparagine synthetase) were observed between two parents (Y11 and GH998). Phylogenetic analysis in *Arabidopsis thaliana* identified two closely related homologs, *AT1G68570* (*AtNPF3.1*) and *AT5G65010* (*ASN2*), which share 72.3 and 87.5% amino acid similarity with *LOC_Os06g15370* and *LOC_Os06g15420*, respectively. Taken together, our results suggested that *qNUE6* is a possible candidate gene for NUE in rice. The fine mapping and candidate gene analysis of *qNUE6* provide the basis of molecular breeding for genetic improvement of rice varieties with high NUE, and lay the foundation for further cloning and functional analysis.

## Introduction

Nitrogen, one of the most demanding mineral elements in plants, is also the most common limiting factor for plant growth in nature. Nitrogen deficiency can cause yellow leaves, dwarf plants, fewer tillers and lower grain yields during the process of rice growth and development. In agricultural production practices, substantial increases in nitrogen fertilizer application has been one of the important ways to increase rice yield (Mulvaney et al., 2009), and it results in agroecological environmental pollution and climate change etc. (Matson et al., 2002; Guo et al., 2010; Shen et al., 2014; Wang Y. et al., 2016). Therefore, there is an urgent need to understand the genetic mechanisms underlying NUE and improve rice varieties NUE via genotype selection (Zeigler and Mohanty, 2010; Mcallister et al., 2012).

The previous studies suggested that NUE is a quantitative trait, being controlled by major genes and minor effects QTLs. The cloning and functional analysis of some genes or QTLs has provided an important basis for understanding the molecular mechanisms underlying rice NUE. Nitrate is the main inorganic nitrogen for its acquisition, transport, assimilation in plants. Members of nitrate/peptide (NTR/PTR) transporter family are the low-affinity nitrate transporters. *NRT1.1*, also known as *CHL1*, was initially identified in *Arabidopsis thaliana* (Tsay et al., 1993). In rice, Lin et al. (2000) cloned *OsNRT1* gene and found that it was homologous to *Arabidopsis thaliana AtNRT1* gene and encodes a low affinity nitrate transporter. Hu et al. (2015) showed that *NRT1.1B* had a nitrate-transporting activity at low and high nitrate ion concentrations. Another nitrate transporter, NRT2, is a high-affinity nitrate transporter but can't transfer ${\text{NO}}_{3}^{-}$ independently. *OsNAR2.1*, a partner protein for the high-affinity nitrate transporter, is able to interact with *OsNRT2.1, OsNRT2.2*, and *OsNRT2.3*, and can enhance nitrate uptake by rice roots at different nitrate supply levels (Yan et al., 2011). Chen et al. (2017) indicated that rice ${\text{NO}}_{3}^{-}$ uptake, yield and NUE were improved by increased *OsNAR2.1* expression via its native promoter. Fan et al. (2016) found that *OsNRT2.3b* was able to increase the pH-buffering capacity of the plant, increasing the uptake of N, Fe, and P, improving NUE and grain yield.

In addition to nitrate, ammonium nitrogen is another main nitrogen source for plant growth. A large number of ammonium transporter (AMT) genes have been identified in rice. Among them, *OsAMT1;1, OsAMT1;2*, and *OsAMT1;3* are the major ammonium transporters that absorb ${\text{NH}}_{4}^{+}$ (Sonoda et al., 2003; Ferreira et al., 2015; Yang et al., 2015). Asparagine synthetase is the major enzyme that assimilates ${\text{NH}}_{4}^{+}$ in rice. *OsAS1* gene is responsible for expressing AS when ammonium is supplied to roots, being involved in primary assimilation of ${\text{NH}}_{4}^{+}$ in roots (Ohashi et al., 2015). Furthermore, Sawaki et al. found that *NIGT1* was a nitrate-induced but self-inhibited transcriptional repressor that played a pivotal role in the response of rice to nitrogen (Sawaki et al., 2013). Sun et al. (2014) mapped and cloned a major QTL for NUE in rice, *qNGR9*, which changed the response of rice to nitrogen by regulating the activity of a G protein. Zhang et al. (2015) identified a major QTL on chromosome 12, *TOND1*, and found that its over-expression enhanced the tolerance of rice to nitrogen deficiency.

In nature, a majority of agronomically important crop traits are quantitative (Paterson et al., 1988). QTL mapping is a highly effective approach for genetic dissection of quantitative traits and provides a starting point for map-based cloning of related genes and marker-assisted selection (Xu et al., 2015). However, the traditional QTL analysis is labor-consuming and costly (Salvi and Tuberosa, 2005). BSA has been applied to rapidly identify the molecular markers closely linked with QTLs or genes by genotyping only two bulked DNA samples from two populations with 20–50 individuals from each, showing extreme opposite trait values for a given phenotype in a segregating progeny (Michelmore et al., 1991). With the development of the next-generation sequencing technology, WGS has been widely applied in genotyping. By combining both BSA and WGS, QTLs for important agronomic traits of crops have been rapidly identified (Takagi et al., 2013). At the present, many genes and QTLs of plants have been mapped through QTL-seq (Takagi et al., 2013, 2015; Liang et al., 2016; Wang H. et al., 2016; Wang Y. et al., 2016; Pandey et al., 2017; Song et al., 2017).

In this study, pools of low and high bulk samples (*n* = 30, each group) were constructed from 280 F_2_ individuals derived from the cross between GH998 (high NUE) and NIL-13B4 (low NUE and derived from “GH998 × Y11”) plants, and then used to detect the regions in the rice genome harboring major QTLs for NUE by BSA, WGS, and single nucleotide polymorphism index (SNP-index) methods. The results were further confirmeded by substitution mapping and qRT-PCR.

## Materials and methods

### Materials and phenotypic identification

In this study, the wild rice Y11 (six generations of single plant bagging selfing) was selected as the donor and the elite rice variety GH998 was selected as the recipient. From the fall 2007, the methods of crossing, back-crossing and marker-assisted selection were adopted. A set of near isogenic lines (BC_4_F_6_) was bred. Then we selected the low NUE near-isogenic lines NIL-13B4 (8.9% genomes were derived from Y11; Supplementary Figure S1) to cross with GH998. In 2014, the F_1_ population was grown at the experimental station (Nanning, 22.85°N, 108.26°E) at Rice Research Institute of Guangxi Academy of Agricultural Sciences, was self-pollinated to generate F_2_ lines that were subsequently used as mapping populations. A total of 280 F_2_ individuals were selected and self-pollinated to generate 280 F_2:3_ families (Zhang and Xu, 2004). The soil in the experimental station is of weak acidity with pH = 6.48, containing 0.12% total phosphorus, 0.11% total phosphorus, 1.78% total potassium, 90.50 mg kg^−1^ available nitrogen, 34.00 mg kg^−1^ available phosphorus and 198.50 mg kg^−1^ available potassium. The field was assigned into groupI(urea, 0 kg.hm^−2^) and groupII(urea, 326.1 kg.hm^−2^). In addition, the potassium chloride at 166.7 kg.hm^−2^ and phosphorus pentoxide at 833.3 kg.hm^−2^ were applied in both groupI and groupII. Plants were transplanted in the plots of 20 m^2^ with the line spacing of 23.1 cm and plant spacing of 13.2 cm with three replicates.

After the rice grains had ripened, 10 representative plants for each replicate were cut along the ground and their stems, leaves and seeds were collected and incubated for heating at 105°C for 30 min followed by incubation for s at 75°C 4 day to constant weight. After being weighted using an electronic balance with resolution of 0.001 g, the total nitrogen content of plant was determined using semi-micro Kjeldahl method (Yoshida et al., 1976a). The NUE was calculated using the following equation (Wei et al., 2012):

$$\begin{array}{l} \text{NUE}\left(\text{\%}\right)=\left(\text{T}{\text{N}}_{\text{F}}-\text{T}{\text{N}}_{0}\right)/\text{N}\times 100 \end{array}$$

TN_0_ is the total nitrogen contents of plants in the non-nitrogen fertilizer treatment group;

TN_F_ is the total nitrogen contents of plants in the nitrogen fertilizer treatment group;

N is the total amount of nitrogen applied.

### DNA isolation and analysis of WGS data

Youngth and healthy leaves of two parents and F_2_ individuals were collected at the tillering stage and stored at −80°C freezer. Their genomic DNA was extracted using CTAB method with modification (Murray and Thompson, 1980). The purity and integrity of each DNA sample were determined by agarose gel electrophoresis. DNA samples of 30 plants with extremely lowNUE were mixed with equal amounts and used as L-pool (30 F_2_ progeny NUE range from 3.6 to 9.8%) and DNA samples of 30 plants with extremely high NUE were mixed with equal amounts and used as H-pool (30 F_2_ progeny NUE range from 26.8 to 34.4%). DNA samples isolated from Y11 and GH998 plant leaves, and the two DNA pools, were randomly fragmented using a covaris crusher to 350 bp, and subjected to terminal repair, polyA tailing, sequencing adaptor ligation, purification and PCR amplification. The sequencing libraries were constructed, which were sequenced on the IlluminaHiSeq2500 platform with sequencing depth of 20× for parental plants and 30× for each pool.

The calculation of SNP-index is a statistical method for SNP in the pool. The principle is that the sequencing reads are used to statistically count the number of reads at certain each base locus with same or different bases to one of its parent or reference genome and the percentages of reads with different bases are caculated, that is, the SNP-index at this base locus. For projects with two sub-pool data, the loci with SNP-index < 0.3 in both pools were filtered out. The average of the filtered SNP-index for 5 kb bases at a window of 50 kb is considered as the SNP-index of the window. The SNP-index of the two pools is calculated according to the above method, and their difference is calculated as the Δ(SNP-index). The difference in SNP-index between the two pools is calculated as Δ(SNP-index) = SNP-index (extreme trait B) -SNP-index (extreme trait A). A thousand replacement tests are performed and the 95% confidence level is selected as the threshold for screening. At the 95% confidence level, a window larger than the threshold is selected as the candidate interval (Takagi et al., 2013).

### QTL analysis with SSR and InDel marker

A total of 24 simple sequence repeat (SSR) markers covering the chromosome 6 were used for mapping genotypes in the 60 F_2_ individuals and gained nine recombinants between the two markers RM539 and RM136 (Supplementary Table S1). These recombinants were used for substitutional mapping with InDel markers (Maeda et al., 2014; Oikawa et al., 2015). Additional InDel markers were designed by us. In detail, the genome sequence of *Oryza sativa* L. ssp. *japonica* cv. Nipponbare (ftp://ftp.ensemblgenomes.org/pub/plants/release-30/fasta/oryza_sativa/Dna/) was used as a reference sequence, and the WGS sequences of Y11 and GH998 were compared using software BWA to find out the InDel difference between the two parents (Y11 and CH998). In addition, the 500 bp sequence framing around the loci were extracted using the self-made pearl language script and the InDel markers with good polymorphism and strong specificity between the two varieties and the two pools were designed using software Primer 5.

### Candidate genes and gene ontology (GO) enrichment analysis

Gene prediction and annotation within the 8,647,275–8,913,783 bp on the QTL region of chromosome 6 were performed using MSU-RGAP (http://rice.plantbiology.msu.edu/). Based on the WGS data of Y11 and GH998, ANNOVAR was used to detect and annotate SNPs or InDels (Wang et al., 2010). Genes with SNPs causing stop gain or loss, non-synonymous and splicing (the introns are close to exons or intron boundary 2 bp), with InDels causing stop gain or loss, frameshift mutation in their corresponding alleles were selected as the candidate genes. Meanwhile, genes with SNPs or InDels in the promoter region (≤1 kb) from the start codon ATG in their corresponding alleles were also selected as the candidate genes. All candidate genes were analyzed by GO enrichment analysis (http://www.geneontology.org/) based on a Fisher's exact test and a Yekutieli multitest adjustment, using a 5% false positive detection threshold.

### Analysis of expression levels of candidate genes by qRT-PCR

The seeds of GH998, Y11 and NIL-13B4 with the same germination status were selected. They were cultured first in double distilled water adjusted to pH 5.5 with MMES-NaOH at 28°C in a light incubator (13 h light/11 h dark) to three-leaf stage, and then in normal nutrient solution (+N,1 mM NH_4_NO_3_) (Yoshida et al., 1976b). Samples of leaves and stem were collectedat after 48 h. Total RNA was extracted with Trizol method (Invitrogen, Carlsbad, CA, USA). Reverse transcription was performed by One-Step gDNA removal and cDNA synthesis superMix (TransGen, Beijing, China). The Actin3 was used as normalization of expression levels of candidate genes with primers F: CCACTATGTTCCCTGGCATT and R: GTACTCAGCCTTGGCAATCC (Sun et al., 2014). The qRT-PCR reaction system was consisted of 2× TransStart SYBR Green Master Mix 10 μl, the forward primer 1 μl, the reverse prime1 μl, template cDNA 1 μl, and double distilled water was added to 20 μl. Reaction program was set as follows: pre-denaturation at 94°C for 5 min, folowed by 35 cycles of 30 s at 94°C, 30 s at 55°C, 1 min at 72°C. The qRT-PCR analysis was performed on AnalytikJena qTOWERE2.2 (AnalytikJena, Germany).

### Phylogenetic analysis of *qNUE6*

MSU-RGAP (http://rice.plantbiology.msu.edu/) contains information about candidate genes for orthologous genes in the other plants. The homologous gene of *Arabidopsis thaliana* were downloaded from TAIR (http://www.arabidopsis.org/index.jsp), while other plants genes were downloaded from Phytozome (https://phytozome.jgi.doe.gov/pz/portal.html). Protein sequence alignment was performed using ClustalW with default parameters. Phylogenetic trees were constructed by MEGA7.0 using Maximum Likelihood method with 1,000 bootstrap replications.

## Results

### Statistical analysis of phenotypes of NUE

**Figure 1:**
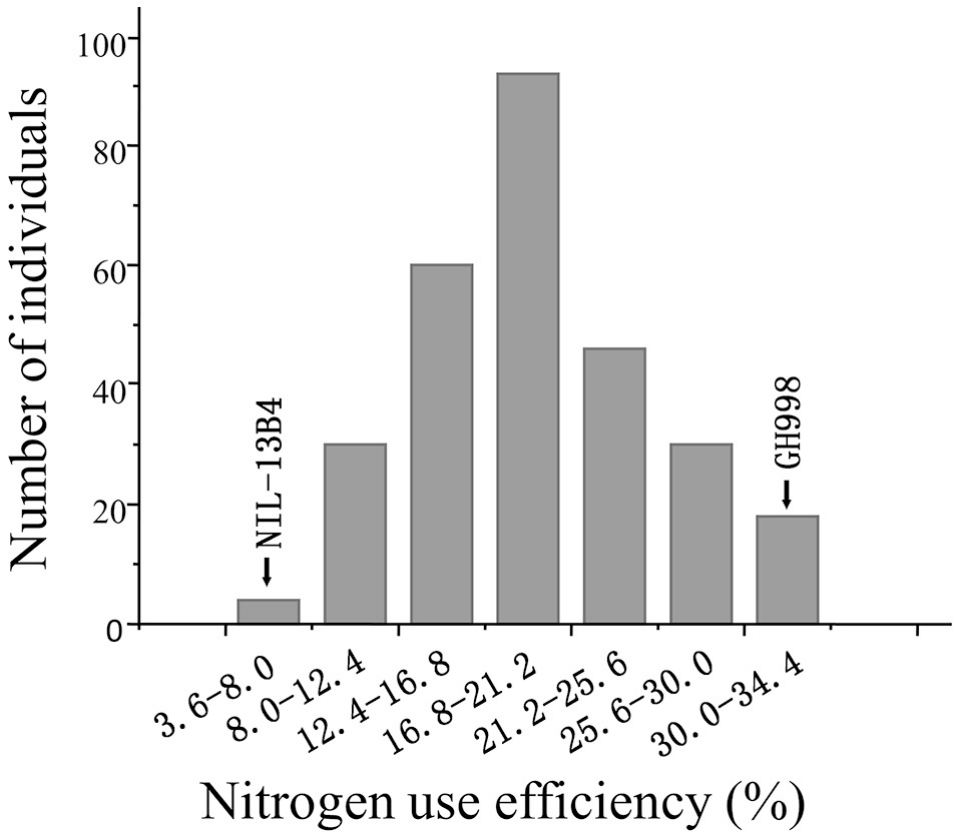
Frequency distribution of nitrogen use efficiency in the F2 population.

The NUE was evaluated for each F_2:3_ families and for two lines (GH998 and NIL-13B4). The average NUE in each individual in F2:3 families represents the NUE in its corresponding F_2_ individual (Zhang and Xu, 2004). As a result, the NUE of the 280 F_2_ lines were fluctuated in the range of 3.64–34.39% with the maximum efficiency being 9.32 times of the minimum efficiency, the mean efficiency of 18.05, the standard deviation of 6.97, and the normality test value of 0.9821, indicating that the phenotype of NUE in F_2_ population is accorded with normal distribution (Figure 1).

### QTL for NUE identified by QTL-seq

Genomic DNA samples of two parents (GH998 and Y11) and the two pools (H-pool and L-pool) were sequenced by IlluminaHiSeq 2500 sequencer and 53.68 Gb raw data were generated. After being filtered, 53.0 GB clean data were obtained (Table 1). These data were end paired and those data with adapter, or nitrogen content exceeding 10% of the total length, more than 50% of bases with quality score Q ≤ 5 were removed.

**Table 1:** The quality of sequencing data.

**Sample**	**Raw base(bp)**	**Clean base(bp)**	**Effective rate(%)**	**Error rate(%)**	**Q20(%)**	**Q30(%)**	**GC content(%)**
Y11	6,867,532,750	6,808,586,750	99.19	0.04	94.48	89.51	42.94
GH998	6,992,487,000	6,925,416,500	99.14	0.04	94.69	89.94	43.38
L-pool	18,695,867,000	18,536,966,000	99.2	0.04	94.64	89.89	43.32
H-pool	21,128,305,000	20,732,080,750	98.12	0.06	92.24	86.2	45.59

**Figure 2:**
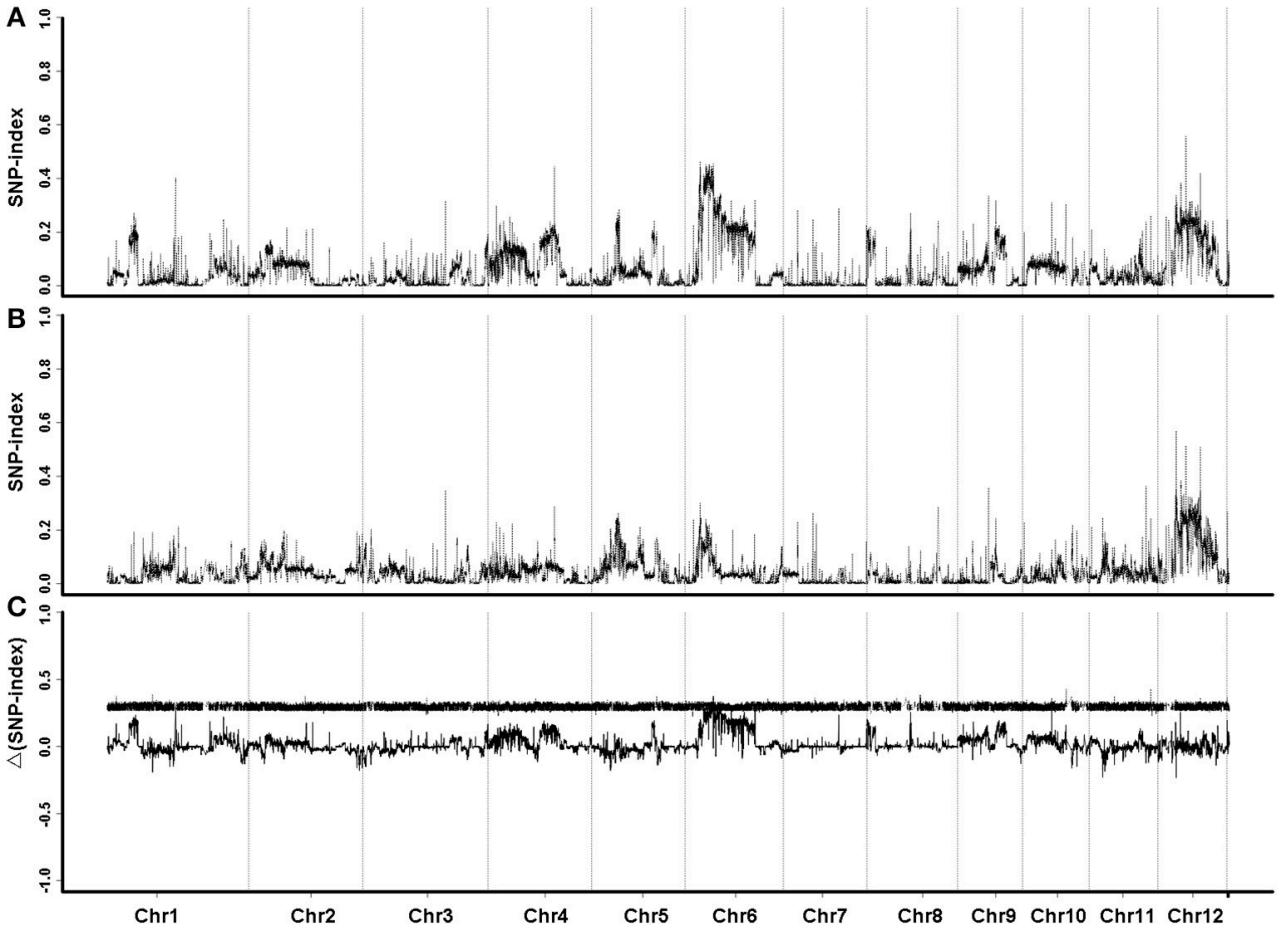
SNP-index graphs of L-pool **(A)**, H-pool **(B)**, and Δ(SNP-index) graph **(C)** from QTL-seq analysis. X-axis represents the position of 12 chromosomes; Y-axis represents the SNP-index. Major QTL is located to chromosome 6.

The sequence data were compared with the reference genome of *Oryza sativa* L. ssp. *japonica* cv. Nipponbare. GH998 had 55,403,332 valid reads, covering 97.76% of the whole genome at average read depth of 17.97×. Y11 had 52,786,428 valid reads, covering 96.91% of the whole genome with average read depth of 17.79× (Table 2). Based on genotyping results, a total of 1,054,326 polymorphic markers were screened from the homozygous SNPs of GH998 and Y11. The SNP-index (the frequency of SNP) of the progeny markers between two parents was calculated using the parental GH998 as a reference genome. Among them, the exact same progeny marker had SNP-index of 0 and the totally different progeny marker had SNP-index of 1 (Takagi et al., 2013). To intuitively reflect the distribution of the progeny SNP-index on the chromosome, the distribution of SNP-index on the chromosome is plotted using 50 kb as the window and average SNP-index of every step of 5 kb. By comparing the SNP-index of low and high NUE pools and analyzing the window above the threshold at 95% confidence level, we found an unbalanced SNP between 6,099,043–8,940,631 bp on chromosome 6 (Figure 2). In this region, the SNP-index of the low pool was greater than or equal to 0.7, and the high pool was lower than or equal to 0.3. These results indicated that the low pool individuals contained the same fragment of Y11 were between 6,099,043 and 8,940,631 bp on chromosome 6 of rice. The high pool individual contained the same fragments of GH998 in the same region. Meanwhile, the Δ(SNP-index) of this region was greater than the threshold at 95% confidence level. Therefore, the region from 6099043 to 8940631 bp is likely the locus controlling NUE in rice, which was named *qNUE6*.

**Table 2:** Statistical analysis of sequencing depth and coverage.

**Sample**	**Mapped reads**	**Total reads**	**Mapping rate(%)**	**Average depth(×)**	**Coverage at least 1 × (%)**	**Coverage at least 4 × (%)**
Y11	52,786,428	54,468,694	96.91	17.79	86.61	78.83
GH998	54,162,109	55,403,332	97.76	17.97	88.72	83.07
L-pool	161,503,688	165,856,646	97.38	50.27	93.19	89.46
H-pool	144,233,962	148,295,728	97.26	45.2	92.38	88.81

### Validation of NUE QTL by substitutional mapping

**Figure 3:**
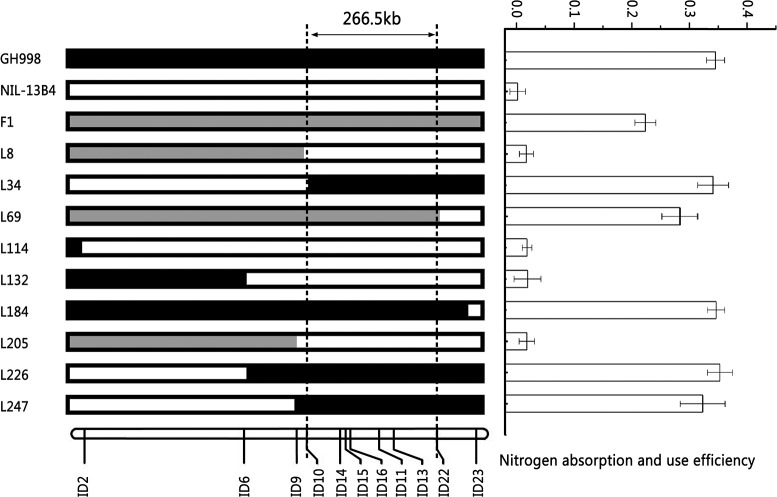
Identification and validation of nitrogen use efficiency QTL qNUE6 on rice chromosome 6. Nine recombinant individuals in the F2 population were used for substitution mapping of qNUE6, which was narrowed down to a 266.5-kb region between the markers ID10 and ID22.

In order to validate the QTL for NUE *qNUE6*, we performed a genome survey using SSR markers covering the chromosome 6. By analysis, the QTL *qNUE6* was narrowed between markers RM539 and RM136 (Supplementary Table S2). We also found nine recombinants between the two markers RM539 and RM136. Subsequently, we designed 20 pairs of InDel markers based on the re-sequencing results of Y11 and GH998. Among them, 11 pairs of InDels markers were found to have good polymorphism and strong specificity (Supplementary Table S3). The nine recombinants were used for substitution mapping of *qNUE6*. The results indicated that *qNUE6* was narrowed down to a 266.5-kb region between markers ID10 and ID22 (Figure 3), which had a physical distance from 8,647,275–8,913,783 bp on chromosome 6.

### Candidate genes and expression analysis

**Figure 4:**
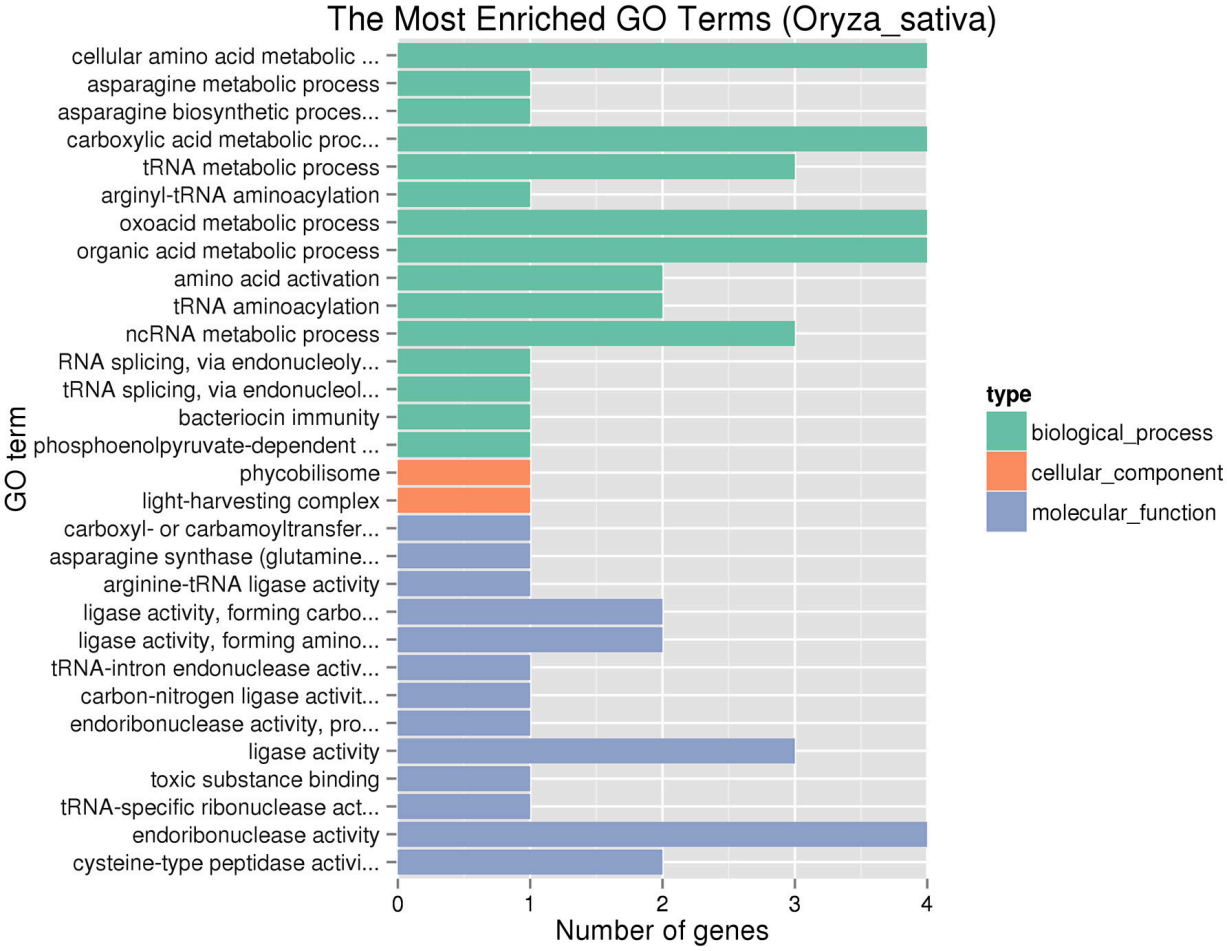
Significantly enriched GO terms of the genes involving SNP or InDel variations.

There are 44 predictive genes (http://rice.plantbiology.msu.edu/) in the region from 8,647,275 to 8,913,783 bp on chromosome 6 (Supplementary Table S4). Among these different loci which contained the candidate region between Y11 and GH998, 692 large-effect SNPs and 20 large-effect InDels were found in a total of 39 genes (Supplementary Tables S5, S6). These genes were identified by GO enrichment analysis in each of the three maincategories (biological processes, molecular function and cellular components). In the significantly enriched GO terms, these terms are involved in biological process (Figure 4), including asparagine metabolic process (GO: 0006528) and asparagine biosynthetic process (GO: 0006529), and TERM asparagine synthetase (glutamine-hydrolyzing) activity (GO: 0004066).

**Figure 5:**
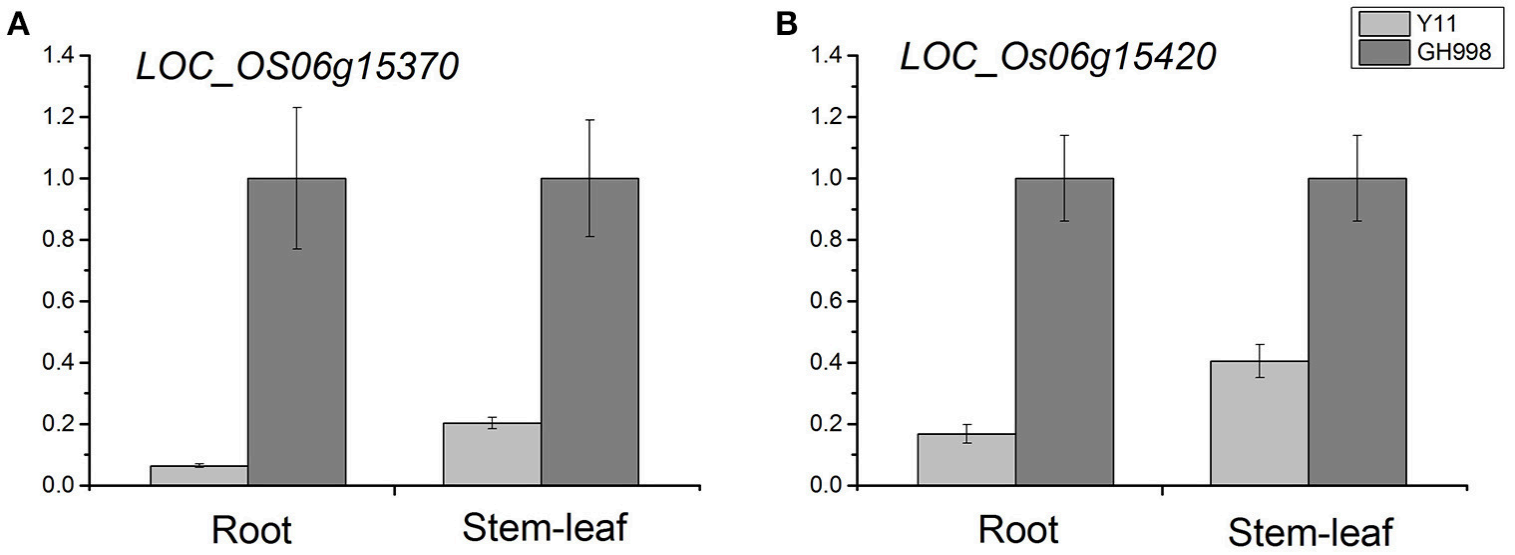
Relative expression of *LOC_Os06g15370* **(A)** and *LOC_Os06g15420* **(B)** after 48 h of trearment with 1 mM NH_4_NO_3_ nutrient solution in GH998 and Y11. The X-axis represents different treatment stage; the Y-axis are scales of relative expression level. Error bars indicate standard deviations of independent biological replicates.

We examined the expression levels of 39 genes in root and stem-leaf using qRT-PCR. Based on the cDNA sequences, we designed 39 primers pairs for qRT-PCR analysis (Supplementary Table S7). The qRT-PCR results showed that no expression levels of 11 genes were detected in root and stem-leaf of Y11 and GH998. Among the 28 genes of normal expression, the expression levels of *LOC_Os06g15370* gene in the root and stem-leaf of Y11 were significantly lower than those in root and stem-leaf of GH998. Similarly, the expression level of *LOC_Os06g15420* in root of Y11was significantly lower than that in GH998, but not obvious expression profile was observed in stem-leaf (Figure 5; Supplementary Figure S2).

### Phylogenic analysis of *qNUE6*

*LOC_Os06g15370* encodes peptide transporter (PTR2) and its conserved domain was analyzed using Batch CD-search (https://www.ncbi.nlm.nih.gov/Structure/cdd/wrpsb.cgi). We found that both *LOC_Os06g15370* and *AT1G68570* in *Arabidopsis thaliana* shared a protein domain of the PTR2 super family. *AT1G68570* was shown to encode a chloroplastic nitrite transporter (Sugiura et al., 2007). *AtNPF3.1* (AT1G68570) was found to be a low-affinity transporter for both nitrate and nitrite, displaying the biophysical characteristics of the known NPF transporters (Pike et al., 2014). Phylogenetic analysis in graminaceous crops revealed that both *GRMZM2G361652* and *Sb10g009530* shared 86.2 and 86.4% similarity in amino acid sequences with *LOC_Os06g15370*, respectively. The *GRMZM2G361652* (PTHR11654:SF178, http://www.pantherdb.org/) in maize putatively encodes nitrite transporter, while *Sb10g009530* (PTHR11654:SF178, http://www.pantherdb.org/) in sorghum encodes nitrate transporter, and both nitrite transporter and nitrate transporter have a domain of PTR2 super family proteins (Supplementary Figure S3).

*LOC_Os06g15420* (*OsAS2*) encodes asparagine synthetase (AS). The results of the study by Ohashi et al. (2015) indicated that OsAS2 mRNA was detectable in the roots, but its expression level was decreased when ${\text{NH}}_{4}^{+}$ was supplied. In *Arabidopsis thaliana, AT5G65010* (*ASN2*) is correlated to ammonium metabolism in higher plants (Wong et al., 2004; Gaufichon et al., 2013), which shares 87.5% similarity in amino acid sequences with *LOC_Os06g15420. OsAS2, ASN2*, and *ASN3* all have a characteristic domain of the protein members in GlmS super family. Phylogenetic analysis in graminaceous crops, indicated that *GRMZM2G074589* and *Sb10g009590* shared 94.4 and 95.6% similarity in amino acid sequences with *LOC_Os06g15420*, respectively (Supplementary Figure S4).

### Evaluation of important agronomic traits of near-isogenic lines of QTL *qNUE6* for NUE in rice

In 2016, heading date, culm length, tiller number, grain number per panicle, 1000-grain weight, and individual yield of NIL-13B4, NIL-13B33 (high NUE and contains *qNUE6*) and GH998 were measured. The results showed that number of tillers was not significantly different among GH998, NIL-13B33, and NIL-13B4, whereas the difference in heading date among them reached a highly significant level and the differences in culm length, 1000-grain weight and single-individual yield among them reached a significant level (Supplementary Table S8).

## Discussion

With the rapid development of molecular biology and genomics, more and more breeders have paid their attention to molecular breeding in plants. Molecular plant breeding can realize the direct selection and effective pyramiding of genes, increase breeding efficiency and shorten the breeding cycles (Mumm and Moose, 2008). In plants, gene mapping, cloning and functional analysis are the foundations for molecular plant breeding. Although many NUE genes or QTLs have been mapped or cloned, the regulatory mechanisms underlying NUE are still complicated, and NUE improvement in rice varieties breeding is very limited (Li et al., 2017). Therefore, we also need to identify new valuable NUE-related QTLs or genes for rice NUE breeding.

In this study, the QTL *qNUE6* were finely mapped by BSA combined with WGS. By further using substitutional mapping with InDel markers, we delimited this QTL in 8,647,275–8,913,783 bp region on chromosome 6. In previous study, researchers have identified several genes or QTLs for NUE on chromosome 6. Song et al. (1996) isolated and identified an aspartate transaminase gene *AspAT3* around 23738029–23738154 bp, which is involved in carbon and nitrogen metabolism in rice. Shan et al. (2005) identified a nitrogen utilization QTL *qNUEp-6* around *Waxy* gene in the 1.76–2.09 Mb region. Tong et al. (2006) detected QTLs related to above ground dry weight and yield within the 28.13–29.63 Mb region under normal and low nitrogen conditions. Wang et al. (2009) detected a novel QTL of effective panicle and yield in the range of 2.29 (RM587)–2.83 Mb (RM510) using rice chromosome fragment segment substitution lines (CSSLs). Liu et al. (2016) used 157 SSR markers to perform genome-wide association analysis of the nitrogen utilization traits of 184 rice cultivars and identified a novel NUE-related locus at SSR marker RM314 (4,845,258–4,845,375 bp). However, these genes or QTLs are not the same loic as *qNUE6*, which is a new QTL.

The region delimited by InDel marker ID10 and ID22 in the reference genome *Oryza sativa* L. ssp *japonica* cv. Nipponbare was predicted to contain 44 genes. Of which, 39 genes were identified as candidates for association with NUE based on large-effect SNPs and large-effect InDels. Among the 39 candidate genes, the expression pattern of *LOC_Os06g15370* and *LOC_Os06g15420* suggested that they might be the candidate genes for NUE in rice. The expression level of *LOC_Os06g15370* was identified significantly lower in Y11than in GH998 by qRT-PCR. It encodes peptide transporter and is highly homologous to the *AtNPF3.1* gene in *Arabidopsis thaliana*, in which, *AtNPF3.1* was found to similarly transport both nitrate and nitrite with low affinity (Pike et al., 2014). Zhao et al. (2010) analyzed 84 PTR family members in rice (OsPTR), and found that the orthologous *OsPTR* and *AtPTR* genes showed the expression profile similar to those of *Os06g15370* and *AT1G68570*. Phylogenetic analysis in graminaceous crops revealed that both *GRMZM2G361652* and *Sb10g009530* shared high similarity in amino acid sequences with *LOC_Os06g15370*, which is a NRT1/PTR FAMILY 3.1 gene (https://phytozome.jgi.doe.gov/pz/portal.html). In rice, three PTR genes have been functionally verified. *LOC_Os03g13274* (*OsNRT1*) encodes low-affinity nitrate transporter and was found to be expressed in stem, cuticle and root hairs (Lin et al., 2000). The results of a study by Hu et al. (2015) showed that variations in the expression of *NRT1.1B* (*LOC_Os10g40600*) largely explained nitrate-use divergence between indica and japonica and that NRT1.1B-indica could potentially improve the NUE of japonica. *SP1* (*LOC_Os11g12740*) is located at the same locus with *OsNPF4.1*, which determines the panicle size (Li et al., 2009). Phylogenetic analysis implied that *SP1* might be a nitrate transporter. However, neither nitrate-transporting activity nor the transporting activities for other compounds could be obtained from it (Léran et al., 2014).

Asparagine synthetase plays important roles in nitrogen metabolism and improvement of NUE in plants (Lam et al., 2003; Gaufichon et al., 2010). In this study, we observed that the expression levels of an asparagine synthetase gene, *LOC_Os06g15420*, were different between Y11 and GH998 in root. It was identified to be related to nitrogen assimilation in the *qNUE6* region. Some asparagine synthetase genes have been found to play an important role in the nitrogen metabolism pathway in maize, *Arabidopsis thaliana* and soybean (Wong et al., 2004; Wan et al., 2006; Cañas et al., 2009; Gaufichon et al., 2013; Han et al., 2015). In phloem sap of rice, glutamine synthetase is the main form of transporter for nitrogen molecules, followed by asparagine synthetase (Hiroaki Hayashi, 1990). Nakano et al. (2000) found that the expression levels of *OsAS* varied in different tissues and different developmental stages of rice and might be closely related to nitrogen assimilation and translocation. Ohashi et al. (2015) showed that both *OsAS1* and *OsAS2* encoded asparagine synthetase and that *OsAS1* was responsible for the initial assimilation of ${\text{NH}}_{4}^{+}$ in root system while *OsAS2* was mainly expressed in rice leaves and leaf sheaths. However, their biological functions in leaves are not clear yet.

Moreover, we identified the agronomic traits between near isogenic lines NIL-13B4 and NIL-13B33 by using the parent GH998 as the control group. The results showed that QTL *qNUE6* might have significant effect on heading date, culm length, 1000-grain weight and individual yield (Supplementary Table S8). Taken together, all of these results lead us to postulate that *qNUE6* can be one of the important candidate genes for NUE in rice.

## Conclusions

In this study, a QTL for NUE, *qNUE6*, was finely mapped through QTL-seq at the region from 8,647,275 to 8,913,783 bp on chromosome 6. *LOC_Os06g15370* and *LOC_Os06g15420* were identified as the candidate genes using gene annotation information, mutation site functional annotation and qRT-PCR. The qRT-PCR analysis showed that the expression levels of *LOC_Os06g15370* and *LOC_Os06g15420* were significantly different between two parents. Phylogenetic analysis revealed that the amino acid sequences of *LOC_Os06g15370* and *LOC_Os06g15420* were highly homologous to those of *AtNPF3.1* gene and *ASN3* gene in *Arabidopsis thaliana*, respectively. The analysis of important agronomic traits in near isogenic lines showed that *qNUE6* might have significant effects on culm length, 1000-grain weight, individual yield, and especially on heading date. However, it is still not clear how *qNUE6* affects the agronomic traits of rice and its biological function has not been further elucidated yet. Thus, further study is needed to elucidate its molecular and biological functions by cloning and transgenic approaches. The identification of *qNUE6* as one of the important candidates for NUE provides an important genetic basis for the improvement of rice varieties with high NUE.

## Accession codes

The sequence data has been deposited in National Center for Biotechnology Information (SRA): SRR5739119, SRR5739120, SRR5739121, SRR5739122, and SRR5739123.

## Author contributions

XY designed and performed the experiment, and wrote the manuscript; XX performed the experiment and drew the graphs; BN, ZZ, and YZ performed the experiment; FX, YW, JG, and GD collected and analyzed data; DL designed and revised the manuscript; all the authors reviewed and approved this submission.

### Conflict of interest statement

The authors declare that the research was conducted in the absence of any commercial or financial relationships that could be construed as a potential conflict of interest.

## Supplementary material

Supplementary Figure S1 Development of NIL-13B4 and F2. **(a)** Schematic to generate NILs (contain NIL-13b4) from “GH998 × Y11.” **(b)** Schematic to generate F2:3 families from “NIL-13B4 × GH998.” **(c)** The genotyping of SNPs in Y11, GH998 and NIL-13B4 by whole genome re-sequencing. **(d)** Graphical genotype of NIL-13B4. Black bar, genomic region from Y11; blue bar, genomic region from GH998.

Supplementary Figure S2 Relative expression of the 28 candidate genes after 48 h of trearment with 1 mM NH_4_NO_3_ nutrient solution in GH998 and Y11.The X-axis represents different treatment stage. The Y-axis are scales of relative expression level. Error bars indicate standard deviations of independent biological replicates.

Supplementary Figure S3 Comparison of amino acid sequences in *LOC_Os06g15370* and homologs. Amino acid sequences of *LOC_Os06g15370* and homologs from *Ananas comosus* (*Aco003000*), *Arabidopsis thaliana* (*AT1G68570*), *Brachypodium distachyon* (*Bradi1g43970*), *Glycine max* (*Glyma.01G081600*), *Musa acuminata* (*GSMUA_Achr5G24480_001*), *Panicum hallii* (*Pahal.D02801*), *Populus trichocarpa* (*POPTR_0010s13620*), *Sorghum bicolor* (*Sb10g009530*), and *Zea mays* (*GRMZM2G361652*) were compared. **(a)** Multiple sequence alignment using ClustalW. **(b)** Molecular phylogenetic analysis by Maximum Likelihood method. Bootstrap analysis was performed with 1,000 replications and the values are expressed as percentages. Scale bar indicates the distance in substitutions per amino acid.

Supplementary Figure S4 Comparison of amino acid sequences in *LOC_Os06g15420* and homologs. Amino acid sequences of *LOC_Os06g15420* and homologs from *Arabidopsis lyrata* (*ARALYDRAFT_908981*), *Arabidopsis thaliana* (*AT5G65010*), *Glycine max* (*GLYMA_18G017200*), *Phaseolus vulgaris* (*PHAVU_001G252200g*), *Populus trichocarpa* (*POPTR_0005s07720g*), *Ricinus communis* (*RCOM_0212760*), *Setaria viridis (evir.9G427500), Sorghum bicolor* (*Sb10g009590*), and *Zea mays* (*GRMZM2G074589*) were compared. **(a)** Multiple sequence alignment using ClustalW. **(b)** Molecular phylogenetic analysis by Maximum Likelihood method. Bootstrap analysis was performed with 1,000 replications and the values are expressed as percentages. Scale bar indicates the distance in substitutions per amino acid.

Supplementary Table S1 Primer sequences used for QTL analysis.

Supplementary Table S2 Genotypes and nitrogen use efficiency of F2 lines.

Supplementary Table S3 Primer sequences used for substitution mapping of QTL qNUE6.

Supplementary Table S4 Forty-four predicted genes of the candidate region on ricce chromosome 6.

Supplementary Table S5 List of genes harboring Large-effect SNPs.

Supplementary Table S6 List of genes harboring Large-effect InDels.

Supplementary Table S7 Primer sequences used for qRT-PCR analysis.

Supplementary Table S8 Agronomic traits of the NILs and GH988. ^*^*P* < 0.05; ^**^*P* < 0.01.
