# Supplementary figures and images for: QTL Mapping by Whole Genome Re-sequencing and Analysis of Candidate Genes for Nitrogen Use Efficiency in Rice
Source: Front Plant Sci. 2017 Sep 21;8:1634. doi: 10.3389/fpls.2017.01634 (PMC5613164; doi:10.3389/fpls.2017.01634)

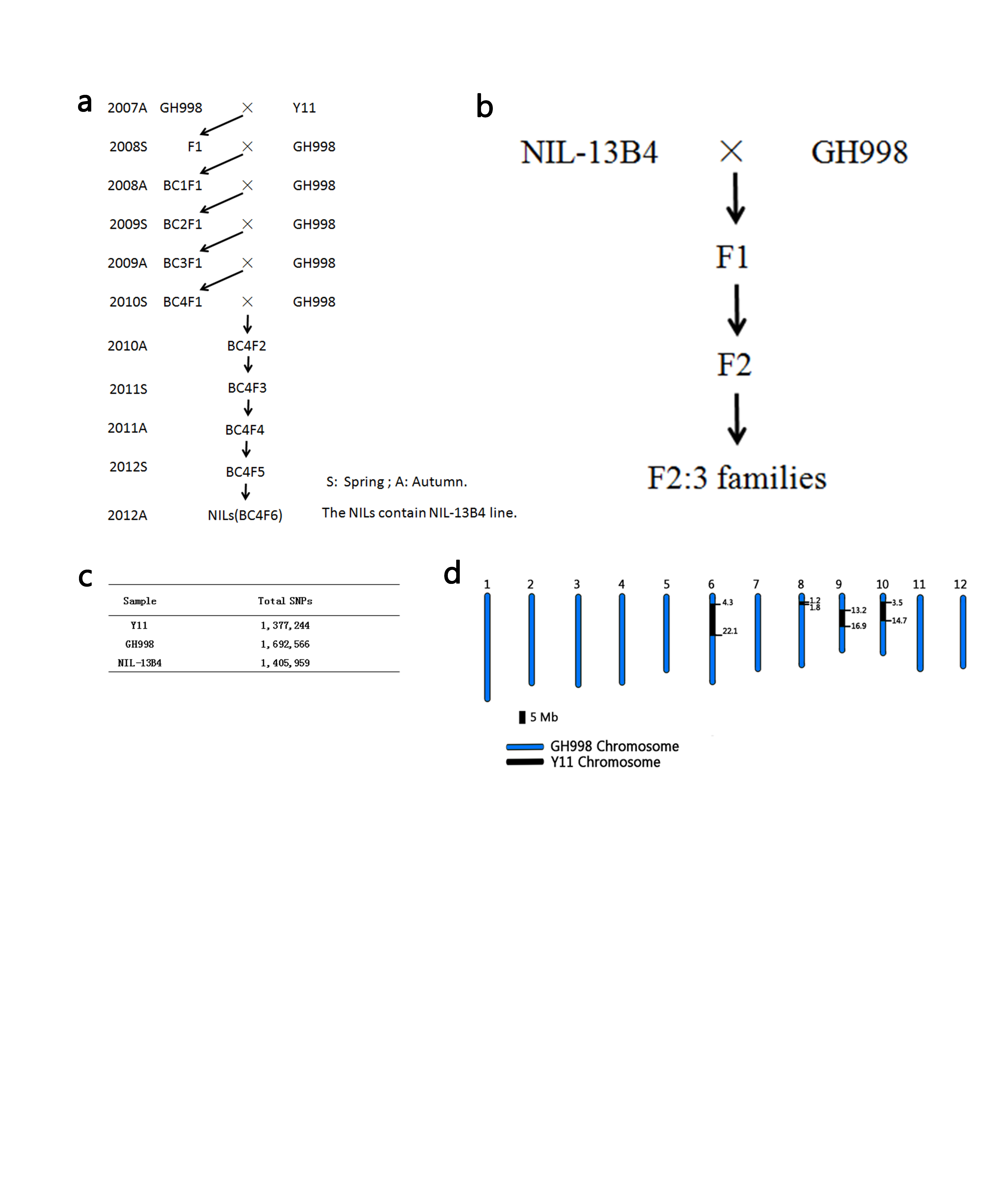

Supplement: Supplementary Figure S1 — Development of NIL-13B4 and F2. (a) Schematic to generate NILs (contain NIL-13b4) from “GH998 × Y11.” (b) Schematic to generate F2:3 families from “NIL-13B4 × GH998.” (c) The genotyping of SNPs in Y11, GH998 and NIL-13B4 by whole genome re-sequencing. (d) Graphical genotype of NIL-13B4. Black bar, genomic region from Y11; blue bar, genomic region from GH998. [file Image1.JPEG]

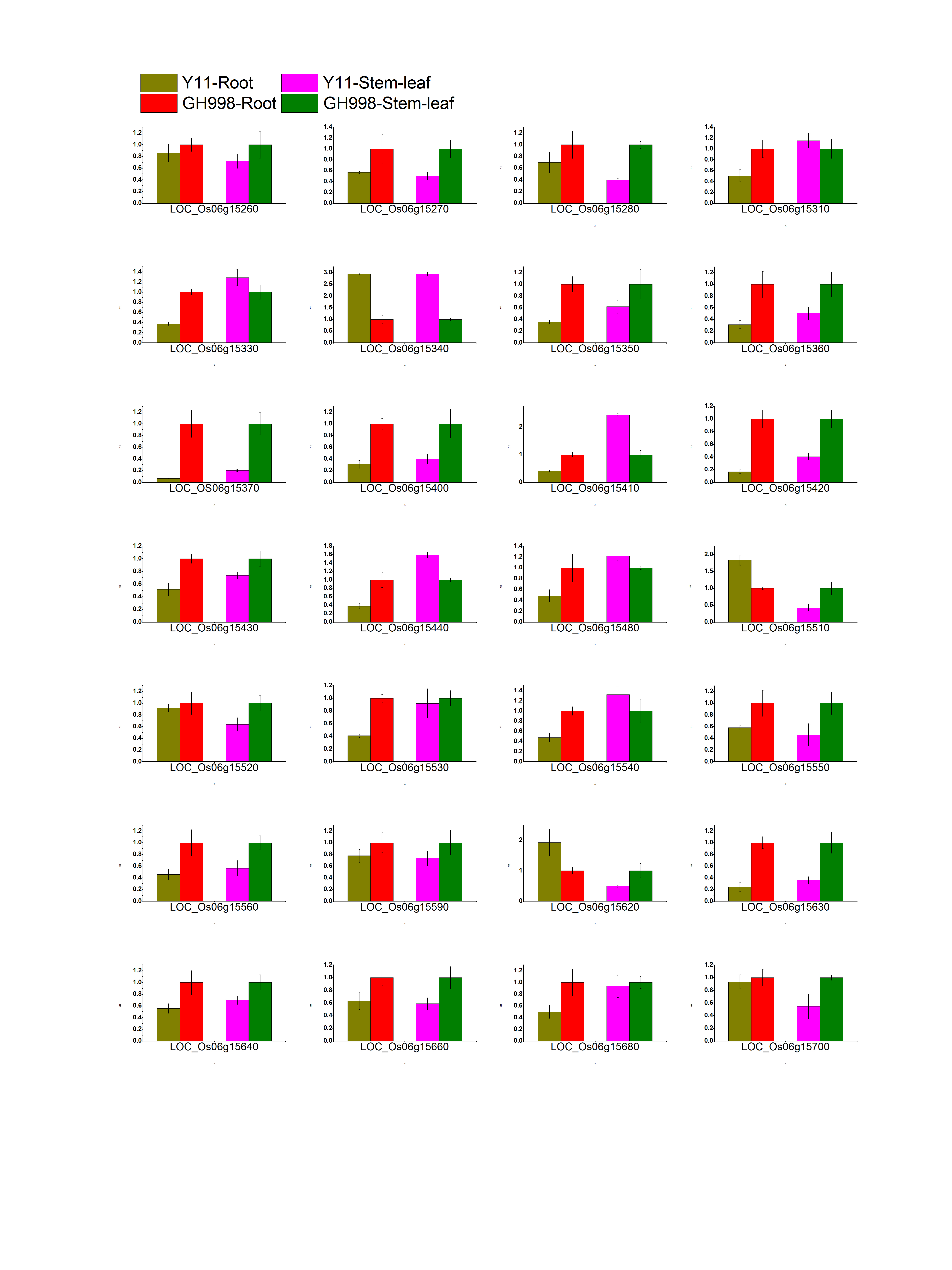

Supplement: Supplementary Figure S2 — Relative expression of the 28 candidate genes after 48 h of trearment with 1 mM NH4NO3 nutrient solution in GH998 and Y11.The X-axis represents different treatment stage. The Y-axis are scales of relative expression level. Error bars indicate standard deviations of independent biological replicates. [file Image2.JPEG]

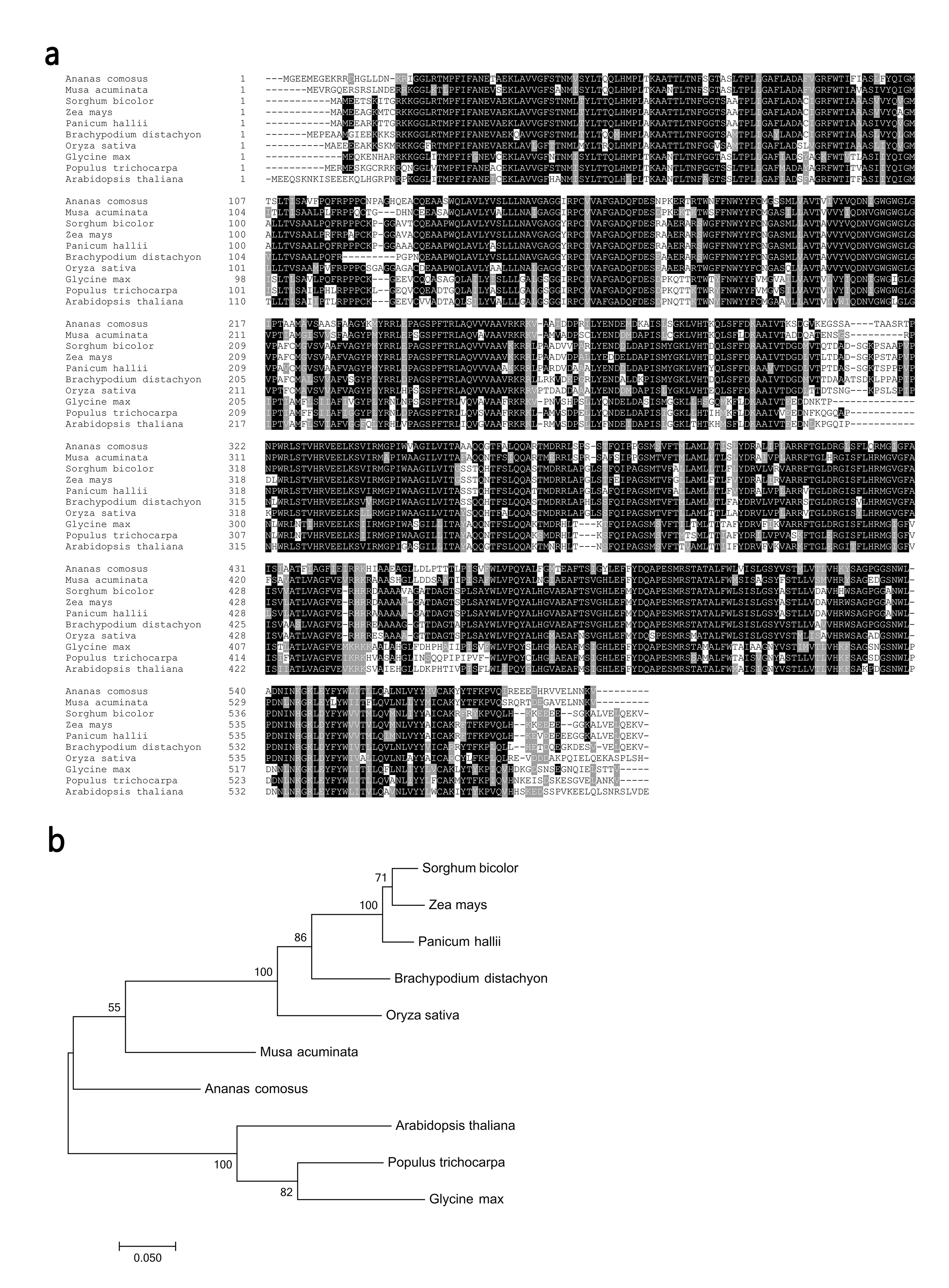

Supplement: Supplementary Figure S3 — Comparison of amino acid sequences in LOC_Os06g15370 and homologs. Amino acid sequences of LOC_Os06g15370 and homologs from Ananas comosus (Aco003000), Arabidopsis thaliana (AT1G68570), Brachypodium distachyon (Bradi1g43970), Glycine max (Glyma.01G081600), Musa acuminata (GSMUA_Achr5G24480_001), Panicum hallii (Pahal.D02801), Populus trichocarpa (POPTR_0010s13620), Sorghum bicolor (Sb10g009530), and Zea mays (GRMZM2G361652) were compared. (a) Multiple sequence alignment using ClustalW. (b) Molecular phylogenetic analysis by Maximum Likelihood method. Bootstrap analysis was performed with 1,000 replications and the values are expressed as percentages. Scale bar indicates the distance in substitutions per amino acid. [file Image3.JPEG]

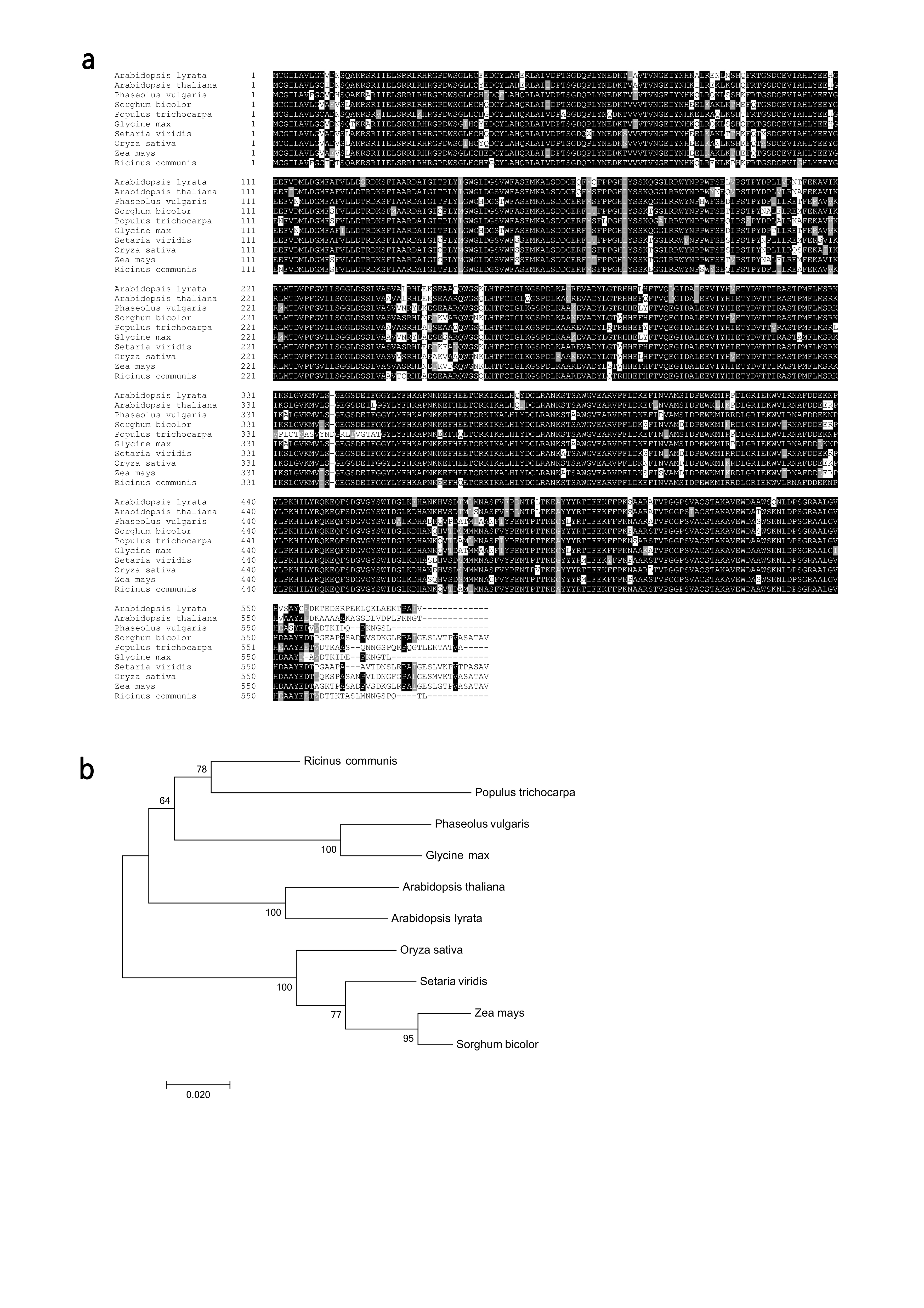

Supplement: Supplementary Figure S4 — Comparison of amino acid sequences in LOC_Os06g15420 and homologs. Amino acid sequences of LOC_Os06g15420 and homologs from Arabidopsis lyrata (ARALYDRAFT_908981), Arabidopsis thaliana (AT5G65010), Glycine max (GLYMA_18G017200), Phaseolus vulgaris (PHAVU_001G252200g), Populus trichocarpa (POPTR_0005s07720g), Ricinus communis (RCOM_0212760), Setaria viridis (evir.9G427500), Sorghum bicolor (Sb10g009590), and Zea mays (GRMZM2G074589) were compared. (a) Multiple sequence alignment using ClustalW. (b) Molecular phylogenetic analysis by Maximum Likelihood method. Bootstrap analysis was performed with 1,000 replications and the values are expressed as percentages. Scale bar indicates the distance in substitutions per amino acid. [file Image4.JPEG]
